# Patterns of Use and Patient-Reported Effects of Cannabinoids in People With PD: A Nationwide Survey

**DOI:** 10.1155/padi/2979089

**Published:** 2025-05-28

**Authors:** Tangui Barré, Géraldine Cazorla, Vincent Di Beo, Fabienne Lopez, Lise Radoszycki, Gwenaëlle Maradan, Christelle Baunez, Patrizia Carrieri

**Affiliations:** ^1^INSERM, IRD, SESSTIM, Sciences Economiques & Sociales de la Santé & Traitement de l'Information Médicale, ISSPAM, Aix-Marseille University, Marseille, France; ^2^Principes Actifs, Lieusaint, France; ^3^Carenity, Paris, France; ^4^ORS PACA, Southeastern Health Regional Observatory, Marseille, France; ^5^La Timone Neuroscience Institute (INT), UMR 7289 CNRS & Aix-Marseille Université, Marseille, France

**Keywords:** cannabidiol, cannabis, pain, Parkinson's disease, sleep disorders

## Abstract

**Background:** People with Parkinson's disease (PD) may use cannabis-based products for symptom management. In France, products containing tetrahydrocannabinol (THC) are prohibited, while cannabidiol (CBD)-products are readily available. However, data on cannabinoid use in French people with PD are lacking.

**Objectives:** To identify correlates of the use of cannabis-based products and to document their patterns of use and perceived effects.

**Methods:** A French nationwide online survey was conducted from May to July 2023. Regression analyses helped identify factors associated with current cannabis and CBD use (regardless of their form). Patterns of use and self-reported effects were also documented.

**Results:** The study sample comprised 1136 participants, with a median age of 68 years. Six percent (5.9%) and 17.9% reported using cannabis and CBD, respectively. Both substances were associated with better knowledge of cannabinoids and a poor self-perceived household economic situation. The most common routes of cannabis administration were oral ingestion (44.8%) and smoking (41.4%); for CBD, they were oral ingestion (82.8%) and smoking (6.4%). Users reported that cannabis and CBD were very effective for sleep disorders, pain, and rigidity/cramps. The satisfaction level for both substances was also high.

**Conclusion:** Cannabis and CBD use among people with PD was associated with better knowledge about cannabinoids and a poor self-perceived household economic situation. Furthermore, users reported high levels of satisfaction for both substances. An enhanced communication with healthcare providers and facilitated access to safe cannabis/CBD products are needed in France to enable people with PD to maximize the benefits of cannabinoids when clinically appropriate.

## 1. Introduction

Parkinson's disease (PD) is characterized by a number of cardinal motor manifestations including bradykinesia, rigidity, and rest tremor [1]. Dopamine replacement strategies are commonly used to treat these symptoms. PD development is also accompanied by several and varied nonmotor symptoms, such as pain and sleep disorders [2], for which treatment options are limited [3]. Long-term dopaminergic treatment may also bring about motor and nonmotor symptoms [4, 5]. All these symptoms weigh heavily on patients' quality of life [6–8]. Compared to healthy persons, people with PD have a lower quality of life in most domains [9].

In the context of suboptimal symptom management, or due to the appeal of more natural treatments [10, 11], PD patients may seek alternative approaches to care [12, 13], including consuming cannabis-based products. In line with findings from subsequent reviews [14, 15], Urbi et al.'s 2021 meta-analysis concluded that in terms of cannabis use in PD patients, “a potential benefit was identified with respect to alleviation of PD–related tremor, anxiety, pain, improvement of sleep quality, and quality of life” [16]. However, only five randomized controlled studies were included in this meta-analysis. Alongside evidence supporting the safety of long-term whole-plant medical cannabis (median tetrahydrocannabinol (THC) level of 10% and median cannabidiol (CBD) level of 4%) [17], the most recent uncontrolled studies have reported benefits on insomnia for a full-spectrum oil (THC: CBD 1000:112 μg/day) [18] and improvement in various symptoms with diverse medical cannabis formulations [19]. A 2023 randomized study supported the safety of an oil-based formulation with varying THC and CBD levels [20]. In contrast, a recent small-sized (*n* = 61) randomized trial found no superiority of a CBD-dominant oil over placebo regarding motor and nonmotor symptoms in people with PD and suggested worsened cognition and sleep [21]. These diverging conclusions contrast with more positive results observed elsewhere from the patients' perspective [13, 22–24].

User-reported effects, profiles, and patterns of use of cannabis-based products in the sphere of self-medication vary according to the product type and are influenced by sociocultural and legal contexts. Unlike countries where medical cannabis is authorized, THC-containing products (over 0.3%) are prohibited in France. The only exception is an ongoing national field experiment aimed at assessing the relevance and feasibility of providing medical cannabis to individuals with chronic severe conditions not sufficiently alleviated by other treatments [25]. PD was not considered to be an eligible indication for enrollment in this experiment.

Unlike THC-containing products, CBD-based products are authorized in France, as in most European countries [26, 27], and are used by a substantial proportion of French adults [28]. This legal framework allows for a wide range of CBD product types to be accessible, including oils, ointments, herbal teas, concentrates, and dried flowers, provided they contain less than 0.3% THC. Therefore, French PD patients seeking cannabis-based symptom relief have two options: obtaining CBD products of uncontrolled quality or purchasing THC-containing cannabis from the black market. The latter likely has high concentrations of THC [29] and is potentially adulterated [30]. On the French black market, cannabis resin is more accessible than herbal cannabis in terms of both availability and price, and in 2018, synthetic cannabinoids were almost exclusively sold online [31]. Notably, among people living with PD, cost did not appear to be a major barrier to self-medication with cannabis or CBD [32].

Due to stigma, including in medical settings [33–39], PD patients may not disclose their use of cannabis-based products to healthcare providers. This is concerning because both THC-containing cannabis and CBD can cause adverse effects and may interact with concurrently prescribed drugs [40].

CBD-based self-medication is rarely studied in PD [41], and no research has examined cannabis use in French PD patients. Identifying PD patients prone to using and understanding their usage patterns could assist healthcare providers in initiating discussions and offering appropriate guidance and harm reduction counseling.

In this context, the present study aimed to identify factors associated with cannabis and CBD use among PD patients living in France, and document their patterns of use and self-reported effects.

## 2. Methods

### 2.1. Study Design and Participants

A cross-sectional online self-administered survey was implemented from 22 May to 14 July 2023 [32]. The two inclusion criteria were being ≥ 18 years old and diagnosed with PD. The link to the survey was displayed on the France Parkinson website. France Parkinson is a national public utility association with 75 local committees and is part of the European PD Association. Invitations to participate in the survey were sent by email to all France Parkinson's contacts (over 35,000 addresses, including approximately 5000 paying members). The association also featured the survey in its newsletter and on its social media. Carenity, a social network for people living with chronic conditions, also invited its members to participate via email and/or private messages on the network's platform (*n* = 1857, including relatives of people with PD). Local associations of people living with PD also helped to disseminate the survey. The survey was closed when a sample size of 1000 participants was reached.

The survey was designed in accordance with the Declaration of Helsinki and approved by the INSERM Ethic Committee (IRB00003888, CD/EB 23-045 dated 4 April, 2023). It was powered by Voxco. Prior to accessing the questionnaire, participants were required to provide informed consent.

### 2.2. Questionnaire and Data Collection

Sociodemographic data collected included gender, age, type of area of residence, educational level, professional situation, and self-perceived household economic status. Health-related data collected included time since PD diagnosis, current intake (yes/no) of dopamine precursors and/or agonists, receiving deep brain stimulation (yes/no), and measures for anxiety and depression from the Generalized Anxiety Disorder Scale-2 (GAD-2) [42], and Patient Health Questionnaire (PHQ-2) [43]. The impact of fatigue was assessed with a question adapted from the Nonmotor Symptoms Scale for PD [44–46]. Disability level was assessed with an item adapted from the PD Composite Scale [47, 48]. Pain was assessed with three questions adapted from the Graded Chronic Pain Scale–Revised [49]. Sleep quality was assessed with a question taken from the Pittsburgh Sleep Quality Index [50].

Cannabinoid knowledge was assessed through four yes/no ad hoc questions (see Supporting Table 1), with a score ranging from 0 (*no correct answer*) to 4 (*four correct answers*). Self-information was assessed with the question “Do you inform yourself on the medical use of cannabis for PD?” Perceived risk of dependence was assessed with the question “In your opinion, how great is the risk of becoming dependent on cannabis?” The participants' position regarding the legal status of cannabis was assessed with two questions: “Are you in favor of alleviating legal restrictions on medical (respectively, nonmedical) use of cannabis in France?”

In the survey, the term “cannabis” referred to cannabis and cannabis-derived products with THC levels above authorized thresholds. The term “CBD” included all CBD-containing products (regardless of their form), with THC levels below authorized thresholds.

“Regular use” (see next section) of either substance was defined as using it at least once a week. Regular users were asked how long they had regularly used (more or less than a year). Users were also asked to specify the type of product they used most often for each of the two substances (e.g., herb and resin) and the primary route of administration for each (e.g., smoked and oral ingestion). Answers were selected from predefined lists. For cannabis and CBD separately, users were asked to assess the effect of each substance on a list of nine symptoms, using the following predefined response options: “I am not concerned by this symptom,” “deterioration,” “no effect on the symptom,” “slight improvement,” “moderate or large improvement,” and “I do not know.” Having experienced adverse effects from using each substance was assessed using a predefined list. Users also indicated whether each substance had an impact on the prescribed medical drugs they were taking. Cannabis users were asked how much attention they paid to the choice of the cannabis strain they used. Users were asked “How do you assess the overall impact of cannabis (respectively, CBD) on your quality of life?” (five possible answers). Finally, user satisfaction was assessed with the question “Would you recommend cannabis (respectively, CBD) as self-medication to a friend in a similar health situation? (Please rate your motivation from 1 to 10).”

### 2.3. Study Outcomes

Two outcomes were defined for the regression analyses: being a cannabis user and being a CBD user. Cannabis (respectively, CBD) use was determined based on respondents selecting any response other than “I do not use it” to the question “How often do you usually use cannabis (respectively, CBD)?.” Possible predefined answers were “I do not use it,” “less than once a week,” “once a week,” “more than once a week but not every day,” “once a day,” “twice a day,” and “three times or more per day.”

### 2.4. Statistical Analyses

All participants who completed the survey questionnaire entirely were included in the analyses. Participants' characteristics were described according to their user status of cannabis and CBD and were compared using chi-square and Mann–Whitney tests for categorical and continuous variables, respectively.

We ran two separate logistic regression models to identify factors associated with cannabis and CBD use. Sociodemographic and health-related variables as well as cannabinoid knowledge (as a continuous variable) were all tested in the models. A *p* value < 0.20 was used as the threshold for identifying eligible variables in the univariable analyses (Wald test). A backward selection procedure was then used to obtain the two multivariable models, with the *p* value threshold for statistical significance set at 0.05.

For comparability purposes, we built final multivariable models for both outcomes based on a common set of variables. Any variable that was maintained in at least one of the previously ran multivariable models was included in the final models, irrespective of their *p* value. Stata/SE 16.1 software (StataCorp LP) was used for all analyses.

## 3. Results

### 3.1. Study Sample Characteristics

The whole study sample comprised 1136 participants, median (interquartile range [IQR]) age was 68.0 [62.0–74.0] years, and 54.8% were men. Six (5.9) percent of the participants used cannabis, 17.9% used CBD, and 4.0% used both (Supporting Figure 1).

Participants' characteristics according to their cannabis and CBD use are shown in Supporting Table 2. Cannabis users, in comparison to nonusers, were more likely to be younger, to be working, to perceive a poor household economic situation, and to have greater cannabinoid knowledge. CBD users, compared to nonusers, were more likely to be working, not to be receiving deep brain stimulation, to experience more impactful chronic pain, and to have greater cannabinoid knowledge.

Cannabis–CBD co-users were more likely to be working, to seek information on medical cannabis, and to be in favor of the alleviation of legal restrictions on nonmedical cannabis use compared to exclusive CBD users. No difference was observed between cannabis–CBD co-users and exclusive cannabis users (data not shown).

Furthermore, 74.4% of the participants were in favor of alleviating legal restrictions on the medical use of cannabis in France, with higher proportions among cannabis and CBD users than among nonusers.

### 3.2. Factors Associated With Cannabis and CBD Use

Results from the two separate logistic regressions are provided in Supporting Table 3. In the final model for cannabis, after multiple adjustments, cannabis use was associated with not being retired, a poor self-perceived household economic situation, and greater cannabinoid knowledge (Table 1).

In the final model for CBD, after multiple adjustments, CBD use was associated with self-perceived household economic difficulties, not receiving deep brain stimulation, more severe pain, and greater cannabinoid knowledge (Table 1).

As most (68.7%) cannabis users were CBD co-users, we performed a post hoc analysis by merging cannabis and CBD users into a single group of cannabis and/or CBD users. In the final related model, after multiple adjustment, cannabis and/or CBD use was associated with professional situation other than working (e.g., occupational disability) (reference: retirement), poor self-perceived household economic situation, not receiving deep brain stimulation, more severe pain, and greater cannabinoid knowledge (Table 1).

### 3.3. Patterns of Cannabis and CBD Use

The frequency of use and duration of regular use (defined as at least once a week) are separately presented for cannabis and CBD users in Supporting Table 4. Daily users accounted for 23.9% and 33.0% of cannabis and CBD users, respectively. Cannabis users were more likely to have regularly used for more than a year than CBD users (*p* < 0.001, z-proportion test).

**Figure 1:**
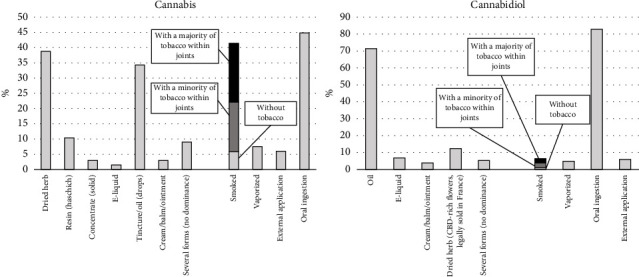
Types of products and routes of administration among cannabis (*n* = 67) and cannabidiol (*n* = 203) users.

The routes of administration for the two substances are presented in Figure 1. The most commonly reported main types of cannabis products were dried herb (38.8%) and tincture/oil (drops) (34.3%), while the most common main routes of cannabis administration were oral ingestion (44.8%) and smoking (41.4%). Among those whose main cannabis products were dried herb or resin, 73.5% reported smoking as the main route of administration. Among cannabis smokers, most mixed it with tobacco.

For CBD users, the most common main products were oil (71.4%) and dried herb (12.3%), while the most common main routes of CBD administration were oral ingestion (82.8%) and smoking (6.4%). Among those whose main CBD products were oil, 95.9% reported oral ingestion as the main route of CBD administration. Among CBD smokers (*n* = 13), the majority (*n* = 11) mixed it with tobacco to some degree.

Among cannabis users, 37.3% paid no attention to the cannabis strain, 26.9% paid moderate attention, and 35.8% paid great attention.

### 3.4. Self-Reported Effects

#### 3.4.1. Beneficial Effects

**Figure 2:**
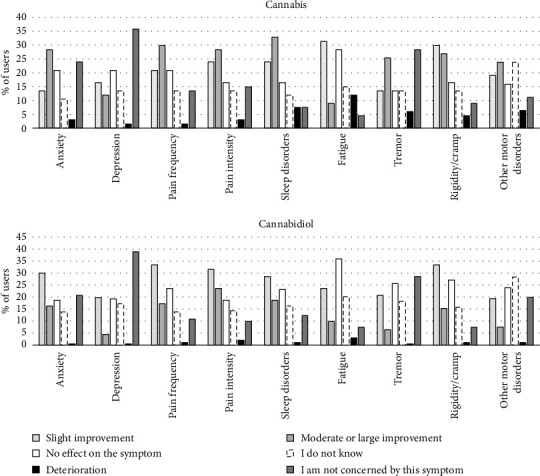
Self-reported effects of cannabis (*n* = 67) and cannabidiol (*n* = 203) use on symptoms.

The self-reported effects of cannabis and CBD on PD symptoms are shown in Figure 2. Cannabis users reported improvements (slight, moderate, or strong) for a median [IQR] number of 3 [1–7] symptoms out of the nine examined. At least one moderate or strong symptom improvement was reported by 80.6% (62.7% for strong improvement) of cannabis users. The most improved symptoms reported by cannabis users were, in descending order: sleep disorders (56.7% of all cannabis users), rigidity/cramps (56.7%), pain intensity (52.2%), pain frequency (50.7%), other motor disorders (42.9%), anxiety (41.8%), fatigue (40.3%), tremor (38.8%), and depression (28.4%). Improvement rates exceeded 60% for the three most-cited symptoms (i.e., sleep disorders, rigidity/cramps, and pain intensity) when participants who declared they were not at all affected by each of these three specific symptoms were excluded.

CBD users reported improvements (slight, moderate or strong) for a median [IQR] number of 3 [2–5] symptoms out of the nine examined. At least one moderate or strong symptom improvement was reported by 87.2% (45.3% for strong improvement) of CBD users. The most improved symptoms reported by CBD users were, in decreasing order: pain intensity (55.2% of all CBD users), pain frequency (50.7%), rigidity/cramps (48.8%), sleep disorders (47.3%), anxiety (46.3%), fatigue (33.5%), tremor (27.1%), other motor disorders (26.9%), and depression (24.1%). Improvement rates exceeded 60% for pain intensity when participants who declared they did not have this symptom were excluded.

The impact of cannabis use on quality of life was reported as “very positive” in 11.9%, “rather positive” in 47.8%, “neutral” in 38.8%, “rather negative” in 0%, and “very negative” in 1.5% of users. For CBD, the impact was reported as “very positive” in 4.9%, “rather positive” in 46.8%, “neutral” in 45.8%, “rather negative” in 2.0%, and “very negative” in 0.5% of users. Median [IQR] satisfaction rates with cannabis and CBD were 7 [5–9] and 7 [5–8], respectively.

#### 3.4.2. Side Effects

For cannabis users, the symptoms that worsened or deteriorated the most with cannabis use were fatigue (11.9%), sleep disorders, other motor disorders, and tremor (between 7.5% and 6.0%). For CBD users, the symptom that worsened or deteriorated the most with CBD use was fatigue (3%) (Figure 2).

**Figure 3:**
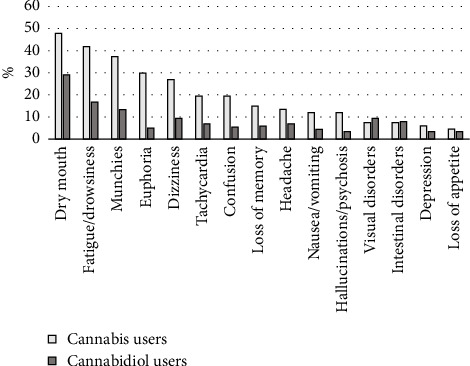
Self-reported side effects in cannabis (*n* = 67) and cannabidiol (*n* = 203) users.

Frequencies of self-reported side effects of cannabis and CBD use are presented in Figure 3. Cannabis users reported a median [IQR] of 2 [0–5] side effects, while CBD users reported a median [IQR] of 0 [0–1] side effects. The most common side effect for both cannabis and CBD was a dry mouth.

Among cannabis users, 80.6% reported that cannabis use had no effect on their use of medical drugs (i.e., for PD or other ailments) consumption; 11.9% reported that they decreased their PD drug intake, while 7.5% decreased their intake of drugs for other ailments. In terms of CBD users, 88.7% reported that it had no effect on their use of drugs (i.e., all ailments); 5.4% reported they decreased their PD drug intake, 1.0% increased it, and 4.9% decreased their use of drugs for other ailments.

## 4. Discussion

To the best of our knowledge, this is the first study to examine cannabis and CBD use among people with PD in France, a country where medical cannabis use is currently not approved. There are two main results: first, cannabis and CBD use were both associated with better knowledge about cannabinoids and a poor self-perceived household economic situation. Second, both cannabis and CBD users reported high effectiveness levels for sleep disorders, pain, and rigidity/cramps, as well as high overall levels of satisfaction with the substances. Dried herb (cannabis) and oil (CBD) were the most commonly used products.

Both cannabis and CBD use were associated with a poor self-perceived household economic situation. One possible reason for this is that that people with PD who need to continue working despite their PD symptoms may more actively seek alternative medicines to better self-manage their symptoms at work. This is partly supported by the association between cannabis use and working conditions in our study. One could also hypothesize that cannabis, which comes from the black market in France, is more easily accessible in deprived socioeconomic areas. In line with findings by Yenilmez et al. [51], both cannabis use and CBD use in our study were associated with better cannabinoid knowledge. We found a similar result regarding acceptability of such products in people living with PD [32]. This association was expected, as self-medication demands active engagement in self-management strategies and access to reliable information sources [52, 53].

CBD use was also associated with greater pain perception. Feeney et al. found pain a common reason for cannabis use [11]. CBD users likely have similar motives, with social media associating CBD with pain relief [54]. A previous study highlighted that CBD users in France frequently reported pain relief as an expected effect [55], reflecting findings from users in Germany [27]. Patients with chronic pain likely view CBD positively [56]. The lack of association between pain and cannabis use in our study may be due to insufficient statistical power.

CBD use was also associated with not receiving deep brain stimulation. The favored target for deep brain stimulation treatment is the subthalamic nucleus, which is a critical node through which nociceptive stimuli enter the basal ganglia circuit [57]. Stimulation of this part of the brain has been shown to reduce pain [58]. Flouty et al.'s meta-analysis reported that deep brain stimulation can improve pain scores by 40% from baseline in PD patients experiencing chronic pain [59]. This would suggest that PD patients in our study who underwent deep brain stimulation surgery suffered less from pain and were therefore less prone to use CBD to treat it.

For both substances, participants reported the most improvement in sleep disorders, pain, and rigidity/cramps. This ranking aligns with patient-reported results from other studies in people with PD using cannabis [11, 23, 24, 51, 60, 61]. To our knowledge, this is the first time that such a ranking has been reported for CBD in people with PD. In their meta-analysis, Urbi et al. pointed out the potential benefits of cannabis for people with PD in terms of improved pain and sleep, as well as tremor [16]. On the contrary, another meta-analysis concluded that no motor symptom improvement could be credited to medical cannabis and its derivatives [62]. In the general population, small positive effects of CBD on pain and sleep have been highlighted or suggested with varying levels of certainty [63–65]. Differences between clinical and patient-reported outcomes in PD [22] can partly be explained by the likelihood that surveys attract active users who experience benefits, explaining the high satisfaction levels for both cannabis and CBD.

In our study, CBD was predominantly ingested orally. However, 41.4% of cannabis users smoked it, mostly mixed with tobacco. Previous studies among people with PD have shown significant variations in routes of administration. In some contexts, cannabis was primarily smoked [61], while in others, other routes of administration were frequent [11, 24, 51, 66]. Although it is difficult to draw definitive epidemiological conclusions about the cannabis smoke's respiratory effects [67], research suggests that cannabis smoke contains higher levels of particulate matter compared to tobacco smoke [68]. Consequently, smoking cannabis is likely to have adverse effects on lung health, particularly when it is obtained on the black market as it may be adulterated. In addition, the co-use of tobacco–cannabis is likely to exacerbate tobacco and cannabis use and/or dependence and reduce the likelihood of cessation [69–71].

In our study, the prevalence of cannabis and CBD use was approximately 6% and 18%, respectively. In Norway, whose legal context resembles France, Erga et al. reported a prevalence of current cannabis use of 11.3% among a sample of 530 people with PD recruited through a process quite similar to ours [23]. However, due to various legal contexts and data collection methodologies, no conclusion can be drawn from comparisons of cannabis use prevalence from other studies [11, 13, 24, 51, 60, 66, 72]. Studies from countries with medical cannabis programs showed that many PD patients still obtain cannabis through nonmedical sources [11, 60, 66].

The difference in prevalence between cannabis and CBD use which we found for France can be attributed to several factors. First, CBD is easily and legally accessible, whereas cannabis use remains *de jure* criminalized, which limits its accessibility and raises legality and safety concerns regarding its supply. Second, CBD is likely to be perceived as less harmful than cannabis. In the French general population, fewer than 20% of adults reported considering CBD as “quite” or “very harmful” [28].

Most cannabis users among our participants also used CBD, though not vice versa. This aligns with general population data in France, where cannabis use is associated with CBD use [28]. In addition, regular cannabis users had used it for longer compared to regular CBD users. Taken together, these findings suggest that cannabis users may turn to CBD as they believe it can provide similar health-related benefits with potentially lower associated risks.

Our results have several implications. First, CBD use does not seem uncommon among French people with PD, underscoring the importance for healthcare providers to inquire about CBD use to address symptom management needs and manage potential adverse effects and drug interactions. Given that a large proportion of cannabis users also reported using CBD, initiating discussions about CBD use may serve as a smoother introduction before addressing cannabis use, which is likely to be more sensitive due to its illegal status. Furthermore, a poor self-perceived household economic situation and more severe self-reported pain among PD patients should prompt physicians to explore potential cannabinoid use. Second, most cannabis users in our study smoked it mixed with tobacco, posing significant cancer-related risks [73] and health risks associated with the quality of unregulated products. Initiating discussions about CBD and cannabis use is crucial for implementing harm reduction strategies, such as promoting smoke-free administration methods like vaporizing [74, 75]. Finally, despite legal constraints in France, users perceive cannabis and CBD as effective for managing PD symptoms, highlighting the need to consider PD as an eligible indication for controlled cannabinoid use under medical supervision. Given that CBD use was not uncommon in our study and that CBD products are already available in various types of retail outlets in France, there should be pharmaceutical-grade, standardized, and easily identifiable CBD products available in pharmacies. Such a measure would prevent patients from purchasing products with uncertain cannabinoid content or potential contaminants [76–81] and would help physicians better assess the doses ingested, thereby improving patient monitoring.

The study's strengths include pioneering documentation of cannabinoid behaviors among people with PD in France, separate documentation of CBD use and effects from cannabis use, a robust national sample size, and a comprehensive collection of sociodemographic and health-related variables.

The study also has limitations. The sample may not be representative of all French people with PD, potentially limiting the generalizability of our findings. Specifically, recruitment primarily targeted individuals affiliated with a particular association, possibly overrepresenting those actively seeking information and engaging in community-based interactions. In addition, the online survey format might have favored the participation of individuals with milder disabilities. There was also a slight underrepresentation of men and overrepresentation of younger patients compared to the typical national French PD population [82]. Furthermore, self-administered questionnaires may be subject to desirability bias particularly regarding such a sensitive topic. However, the online format likely mitigated any such bias. Finally, PD diagnosis and severity were not clinically validated in our study, and we were unable to quantitatively assess participants' cannabinoid intake.

To conclude, unlike cannabis use, CBD use was common in our sample of people with PD in France. Both uses were associated with better knowledge of cannabinoids and a poor self-perceived household economic situation. Users commonly reported improvements in sleep disorders, pain, and rigidity/cramps. An enhanced communication with healthcare providers and facilitated access to safe products are needed in France so that people with PD can maximize the benefits of cannabinoids when clinically appropriate.

## Figures and Tables

**Table 1:** Factors associated with cannabis and cannabidiol use (final multivariable binary regression models).

	Cannabis		Cannabidiol		Cannabis and/or cannabidiol	
	aOR (95% CI)	*p* value	aOR (95% CI)	*p* value	aOR (95% CI)	*p* value
Professional situation						
Retired	1		1		1	
Working	2.57 [1.34–4.91]	**0.004**	0.94 [0.57–1.56]	0.809	1.18 [0.74–1.91]	0.484
Other (including occupational disability)	2.63 [1.39–4.95]	**0.003**	1.53 [0.98–2.38]	0.061	1.59 [1.03–2.44]	**0.036**
“Presently, would you say that in your household, financially speaking…?”						
You are ok/you are comfortable	1		1		1	
You just get by	1.24 [0.67–2.31]	0.490	1.22 [0.82–1.82]	0.324	1.16 [0.78–1.71]	0.468
It's difficult to make ends meet/you can't manage without going into debt	2.28 [1.11–4.69]	**0.025**	1.77 [1.02–3.05]	**0.041**	1.99 [1.18–3.34]	**0.010**
Receiving deep brain stimulation						
No	1		1		1	
Yes	0.85 [0.35–2.10]	0.725	0.39 [0.19–0.83]	**0.014**	0.42 [0.21–0.84]	**0.014**
“Over the past 3 months, what number best describes your level of pain on average (0–10)?” per one-unit increase	1.03 [0.94–1.14]	0.492	1.09 [1.02–1.16]	**0.008**	1.07 [1.01–1.14]	**0.021**
Cannabinoid knowledge (0–4)^1^ per one-unit increase	1.53 [1.23–1.90]	**< 0.001**	2.26 [1.93–2.65]	**< 0.001**	2.25 [1.94–2.61]	**< 0.001**

*Note:* Bold indicates *p* values ≤ 0.05.

Abbreviation: CI, confidence interval.

^1^Scoring based on the correctness of four ad hoc questions.

## Data Availability

The data that support the findings of this study are available on request from the corresponding author. The data are not publicly available due to privacy or ethical restrictions.

## Supporting Information

Supporting Information 1 Supporting Figure 1. Distribution of participants according to cannabis and cannabidiol use.

Supporting Information 2 Supporting Table 1: Questions assessing participants' knowledge about cannabis and cannabinoids.

Supporting Information 3 Supporting Table 2: Study sample characteristics according to cannabis and cannabidiol use.

Supporting Information 4 Supporting Table 3. Factors associated with cannabis and cannabidiol use (multivariable binary regression models).

Supporting Information 5 Supporting Table 4. Frequency and duration of use for cannabis and cannabidiol.
