# Supplementary figures and images for: Patterns of Use and Patient-Reported Effects of Cannabinoids in People With PD: A Nationwide Survey
Source: Parkinsons Dis. 2025 May 28;2025:2979089. doi: 10.1155/padi/2979089 (PMC12136873; doi:10.1155/padi/2979089)

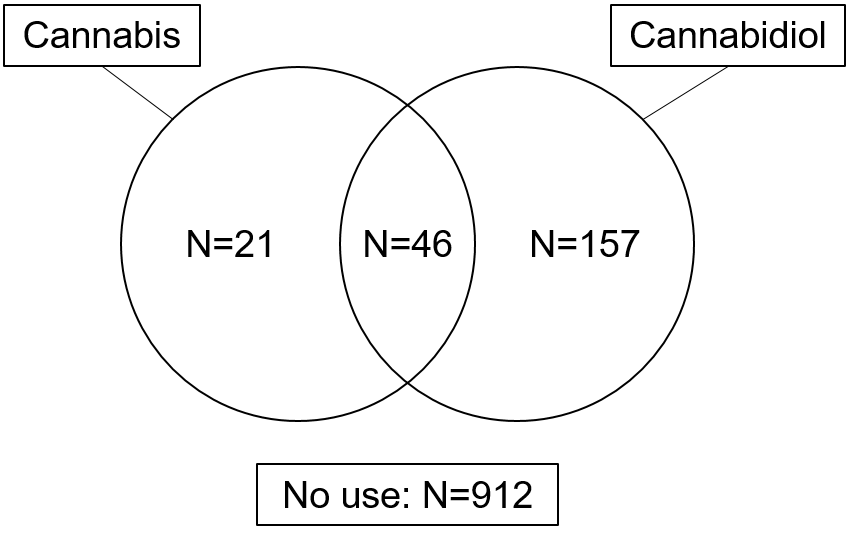

Supplement: Supporting Information 1 — Supporting Figure 1. Distribution of participants according to cannabis and cannabidiol use. [file 2979089.f1.png]
