# Supplementary material for: Patterns of Use and Patient-Reported Effects of Cannabinoids in People With PD: A Nationwide Survey
Source: Parkinsons Dis. 2025 May 28;2025:2979089. doi: 10.1155/padi/2979089 (PMC12136873; doi:10.1155/padi/2979089)
Supplement: Supporting Information 2 — Supporting Table 1: Questions assessing participants' knowledge about cannabis and cannabinoids. [file 2979089.f2.docx]

**Supplementary Table 1: Questions assessing participants’ knowledge about cannabis and cannabinoids**

|  | **Possible answers (correct answers are underlined)** | **Whole sample (n=1136)**  **% of correctness** | **Non-users (n=912)**  **% of correctness** | **Cannabis and/or cannabidiol users (n=224)**  **% of correctness** | **Cannabis users (n=67)**  **% of correctness** | **Cannabidiol users (n=203)**  **% of correctness** |
| --- | --- | --- | --- | --- | --- | --- |
| Cannabidiol (CBD) is an active ingredient naturally present in the cannabis plant | True/False/I do not know | 73.5 | 68.8 | 92.9 | 94.0 | 92.6 |
| Cannabidiol (CBD) can impair some mental abilities (i.e., it can induce a *high*) | True/False/I do not know | 40.9 | 33.2 | 71.9 | 64.2 | 71.9 |
| The active ingredient tetrahydrocannabinol (THC) can impair some mental abilities (i.e., it can induce a *high*) | True/False/I do not know | 40.9 | 33.5 | 62.5 | 61.2 | 62.6 |
| Cannabidiol (CBD) is illegal in France | True/False/I do not know | 56.4 | 50.9 | 79.0 | 62.7 | 81.3 |
