# Supplementary material for: Patterns of Use and Patient-Reported Effects of Cannabinoids in People With PD: A Nationwide Survey
Source: Parkinsons Dis. 2025 May 28;2025:2979089. doi: 10.1155/padi/2979089 (PMC12136873; doi:10.1155/padi/2979089)
Supplement: Supporting Information 3 — Supporting Table 2: Study sample characteristics according to cannabis and cannabidiol use. [file 2979089.f3.docx]

**Supplementary Table 2. Study sample characteristics according to cannabis and cannabidiol use**

| **Characteristics (% of ‘do not know’)** | **Whole study sample (n=1136, %)** | **Cannabis non-users (n=1069 94.1%)** | **Cannabis users^1^**  **(n=67**  **5.9%)** | **p-value non-users vs. cannabis users^2^** | **Cannabidiol non users**  **(n=933**  **82.1%)** | **Cannabidiol users^1^**  **(n=203**  **17.9%)** | **p-value non-users vs. cannabidiol users^2^** |
| --- | --- | --- | --- | --- | --- | --- | --- |
| **Gender** |  |  |  | 0.398 |  |  | 0.141 |
| Men | 623 (54.8%) | 584 (54.6%) | 39 (58.2%) |  | 522 (55.9%) | 101 (49.8%) |  |
| Women | 511 (45.0%) | 485 (45.4%) | 26 (38.8%) |  | 411 (44.1%) | 100 (49.3%) |  |
| Other^3^ | 2 (0.2%) | 0 (0%) | 2 (3.0%) |  | 0 (0%) | 2 (1.0%) |  |
| **Age (in years, median [IQR])** | 68.0 (62.0, 74.0) | 68.0 (62.0, 74.0) | 64.0 (54.0, 70.0) | 0.001 | 68.0 (62.0, 74.0) | 67.0 (60.0, 73.0) | 0.106 |
| **Area of residence** |  |  |  | 0.929 |  |  | 0.544 |
| Rural area | 376 (33.1%) | 353 (33.0%) | 23 (34.3%) |  | 303 (32.5%) | 73 (36.0%) |  |
| Medium-sized city | 484 (42.6%) | 455 (42.6%) | 29 (43.3%) |  | 404 (43.3%) | 80 (39.4%) |  |
| Large city (>200 000 inhabitants) | 276 (24.3%) | 261 (24.4%) | 15 (22.4%) |  | 226 (24.2%) | 50 (24.6%) |  |
| **Educational level** |  |  |  | 0.475 |  |  | 0.063 |
| < upper secondary  school certificate | 282 (24.8%) | 267 (25.0%) | 15 (22.4%) |  | 241 (25.8%) | 41 (20.2%) |  |
| upper secondary  school certificate | 166 (14.6%) | 160 (15.0%) | 6 (9.0%) |  | 143 (15.3%) | 23 (11.3%) |  |
| Tertiary educational diploma (< Master’s degree) | 404 (35.6%) | 377 (35.3%) | 27 (40.3%) |  | 318 (34.1%) | 86 (42.4%) |  |
| Tertiary educational diploma (≥ Master’s degree) | 284 (25.0%) | 265 (24.8%) | 19 (28.4%) |  | 231 (24.8%) | 53 (26.1%) |  |
| **Professional situation** |  |  |  | <0.001 |  |  | 0.006 |
| Retired | 841 (74.0%) | 808 (75.6%) | 33 (49.3%) |  | 706 (75.7%) | 135 (66.5%) |  |
| Working | 153 (13.5%) | 136 (12.7%) | 17 (25.4%) |  | 112 (12.0%) | 41 (20.2%) |  |
| Other (including occupational disability) | 142 (12.5%) | 125 (11.7%) | 17 (25.4%) |  | 115 (12.3%) | 27 (13.3%) |  |
| **“Presently, would you say that in your household, financially speaking…?”** |  |  |  | 0.036 |  |  | 0.209 |
| It’s difficult to make ends meet/You can’t manage without going into debt | 110 (9.7%) | 97 (9.1%) | 13 (19.4%) |  | 84 (9.0%) | 26 (12.8%) |  |
| You just get by | 265 (23.3%) | 248 (23.2%) | 17 (25.4%) |  | 214 (22.9%) | 51 (25.1%) |  |
| You are ok | 445 (39.2%) | 423 (39.6%) | 22 (32.8%) |  | 376 (40.3%) | 69 (34.0%) |  |
| You are comfortable | 316 (27.8%) | 301 (28.2%) | 15 (22.4%) |  | 259 (27.8%) | 57 (28.1%) |  |
| **Time since Parkinson’s disease diagnosis (in years, median [IQR]) (0.3)** | 7.0 (4.0, 11.0) | 7.0 (4.0, 11.0) | 7.0 (4.0, 11.0) | 0.745 | 7.0 (4.0, 11.0) | 6.0 (4.0, 11.0) | 0.097 |
| **Taking dopamine precursors (0.6)** |  |  |  | 0.368 |  |  | 0.459 |
| No | 86 (7.6%) | 79 (7.4%) | 7 (10.4%) |  | 68 (7.3%) | 18 (8.9%) |  |
| Yes | 1043 (92.4%) | 983 (92.6%) | 60 (89.6%) |  | 858 (92.7%) | 185 (91.1%) |  |
| **Taking dopamine agonists (3.0)** |  |  |  | 0.630 |  |  | 0.642 |
| No | 487 (44.2%) | 461 (44.4%) | 26 (41.3%) |  | 397 (43.9%) | 90 (45.7%) |  |
| Yes | 615 (55.8%) | 578 (55.6%) | 37 (58.7%) |  | 508 (56.1%) | 107 (54.3%) |  |
| **Receiving deep brain stimulation** |  |  |  | 0.747 |  |  | 0.042 |
| No | 1046 (92.1%) | 985 (92.1%) | 61 (91.0%) |  | 852 (91.3%) | 194 (95.6%) |  |
| Yes | 90 (7.9%) | 84 (7.9%) | 6 (9.0%) |  | 81 (8.7%) | 9 (4.4%) |  |
| **GAD-2 score (median [IQR])^4^ (7.0)** | 2.0 (1.0, 4.0) | 2.0 (1.0, 4.0) | 2.0 (1.0, 4.0) | 0.748 | 2.0 (1.0, 4.0) | 2.0 (1.0, 4.0) | 0.139 |
| **GAD-2 score ≥ 3^4^ (7.0)** |  |  |  | 0.639 |  |  | 0.377 |
| No | 664 (62.9%) | 622 (62.7%) | 42 (65.6%) |  | 548 (63.5%) | 116 (60.1%) |  |
| Yes | 392 (37.1%) | 370 (37.3%) | 22 (34.4%) |  | 315 (36.5%) | 77 (39.9%) |  |
| **PHQ-2 score (median [IQR]) ^5^ (6.1)** | 2.0 (0.0, 3.0) | 2.0 (0.0, 3.0) | 2.0 (1.0, 4.0) | 0.543 | 2.0 (0.0, 3.0) | 2.0 (1.0, 3.0) | 0.512 |
| **PHQ-2 score ≥ 3^5^ (6.1)** |  |  |  | 0.386 |  |  | 0.616 |
| No | 772 (72.4%) | 728 (72.7%) | 44 (67.7%) |  | 631 (72.0%) | 141 (73.8%) |  |
| Yes | 295 (27.6%) | 274 (27.3%) | 21 (32.3%) |  | 245 (28.0%) | 50 (26.2%) |  |
| **Fatigue as a limit to daily activities**^6^ |  |  |  | 0.623 |  |  | 0.883 |
| Never/ Rarely (< once/week) | 220 (19.4%) | 205 (19.2%) | 15 (22.4%) |  | 184 (19.7%) | 36 (17.7%) |  |
| Regularly (once a week) | 238 (21.0%) | 227 (21.2%) | 11 (16.4%) |  | 194 (20.8%) | 44 (21.7%) |  |
| Often (several times a week) | 373 (32.8%) | 353 (33.0%) | 20 (29.9%) |  | 303 (32.5%) | 70 (34.5%) |  |
| Very often (every day) | 305 (26.8%) | 284 (26.6%) | 21 (31.3%) |  | 252 (27.0%) | 53 (26.1%) |  |
| **Disability level**^7^ |  |  |  | 0.434 |  |  | 0.611 |
| Able to perform daily activity without problems. | 297 (26.1%) | 279 (26.1%) | 18 (26.9%) |  | 245 (26.3%) | 52 (25.6%) |  |
| Limitations in carrying out demanding daily activities or activities requiring fine motor skills. | 446 (39.3%) | 426 (39.9%) | 20 (29.9%) |  | 359 (38.5%) | 87 (42.9%) |  |
| Limitations to perform basic daily activities. | 202 (17.8%) | 188 (17.6%) | 14 (20.9%) |  | 165 (17.7%) | 37 (18.2%) |  |
| Needs help to perform some basic daily activities. | 140 (12.3%) | 130 (12.2%) | 10 (14.9%) |  | 120 (12.9%) | 20 (9.9%) |  |
| Dependent of other persons to perform all basic daily activities. | 51 (4.5%) | 46 (4.3%) | 5 (7.5%) |  | 44 (4.7%) | 7 (3.4%) |  |
| **Over the past three months, how often have you had pain?** ^8^ |  |  |  | 0.367 |  |  | 0.014 |
| Never | 118 (10.4%) | 109 (10.2%) | 9 (13.4%) |  | 105 (11.3%) | 13 (6.4%) |  |
| Some days | 403 (35.5%) | 384 (35.9%) | 19 (28.4%) |  | 335 (35.9%) | 68 (33.5%) |  |
| Most days | 267 (23.5%) | 253 (23.7%) | 14 (20.9%) |  | 223 (23.9%) | 44 (21.7%) |  |
| Every day | 339 (29.8%) | 314 (29.4%) | 25 (37.3%) |  | 261 (28.0%) | 78 (38.4%) |  |
| I do not know^3^ | 9 (0.8%) | 9 (0.8%) | 0 (0%) |  | 9 (1.0%) | 0 (0%) |  |
| **Over the past three months, how often has pain limited your life or work activities?** ^8^ |  |  |  | 0.501 |  |  | 0.201 |
| Never | 236 (20.8%) | 222 (20.8%) | 14 (20.9%) |  | 203 (21.8%) | 33 (16.3%) |  |
| Some days | 492 (43.3%) | 467 (43.7%) | 25 (37.3%) |  | 404 (43.3%) | 88 (43.3%) |  |
| Most days | 219 (19.3%) | 205 (19.2%) | 14 (20.9%) |  | 177 (19.0%) | 42 (20.7%) |  |
| Every day | 169 (14.9%) | 155 (14.5%) | 14 (20.9%) |  | 131 (14.0%) | 38 (18.7%) |  |
| I do not know^3^ | 20 (1.8%) | 20 (1.9%) | 0 (0%) |  | 18 (1.9%) | 2 (1.0%) |  |
| **Chronic pain^9^ (1.1)** |  |  |  | 0.225 |  |  | 0.049 |
| Absent | 521 (46.4%) | 493 (46.6%) | 28 (41.8%) |  | 440 (47.7%) | 81 (40.1%) |  |
| Mild or bothersome | 238 (21.2%) | 227 (21.5%) | 11 (16.4%) |  | 197 (21.4%) | 41 (20.3%) |  |
| High impact | 365 (32.5%) | 337 (31.9%) | 28 (41.8%) |  | 285 (30.9%) | 80 (39.6%) |  |
| **Over the past three months, what number best describes your level of pain on average? (median [IQR])** ^8^ | 5.0 (2.0, 6.0) | 5.0 (2.0, 6.0) | 5.0 (1.0, 7.0) | 0.249 | 4.0 (2.0, 6.0) | 5.0 (3.0, 7.0) | 0.007 |
| **During the past month, how would you rate**  **your sleep quality overall?^10^** |  |  |  | 0.723^11^ |  |  | 0.512 |
| Very good | 87 (7.7%) | 84 (7.9%) | 3 (4.5%) |  | 76 (8.1%) | 11 (5.4%) |  |
| Fairly good | 456 (40.1%) | 429 (40.1%) | 27 (40.3%) |  | 368 (39.4%) | 88 (43.3%) |  |
| Fairly poor | 441 (38.8%) | 415 (38.8%) | 26 (38.8%) |  | 364 (39.0%) | 77 (37.9%) |  |
| Very poor | 152 (13.4%) | 141 (13.2%) | 11 (16.4%) |  | 125 (13.4%) | 27 (13.3%) |  |
| **Cannabidiol knowledge** **(median [IQR])^12^** | 2.0 (1.0, 3.0) | 2.0 (1.0, 3.0) | 3.0 (2.0, 4.0) | <0.001 | 2.0 (1.0, 3.0) | 3.0 (2.0, 4.0) | <0.001 |
| **Do you inform yourself on the medical use of cannabis for Parkinson’s disease?** |  |  |  | <0.001 |  |  | <0.001 |
| Not at all | 409 (36.0%) | 404 (37.8%) | 5 (7.5%) |  | 375 (40.2%) | 34 (16.7%) |  |
| Yes, somewhat | 509 (44.8%) | 470 (44.0%) | 39 (58.2%) |  | 414 (44.4%) | 95 (46.8%) |  |
| Yes, absolutely | 218 (19.2%) | 195 (18.2%) | 23 (34.3%) |  | 144 (15.4%) | 74 (36.5%) |  |
| **In your opinion, how great is the risk of becoming dependent on cannabis?** |  |  |  | <0.001^11^ |  |  | <0.001 |
| There is no risk | 75 (6.6%) | 62 (5.8%) | 13 (19.4%) |  | 54 (5.8%) | 21 (10.3%) |  |
| Slight | 216 (19.0%) | 195 (18.2%) | 21 (31.3%) |  | 168 (18.0%) | 48 (23.6%) |  |
| Moderate | 188 (16.5%) | 167 (15.6%) | 21 (31.3%) |  | 149 (16.0%) | 39 (19.2%) |  |
| Serious | 289 (25.4%) | 282 (26.4%) | 7 (10.4%) |  | 235 (25.2%) | 54 (26.6%) |  |
| Very serious | 95 (8.4%) | 92 (8.6%) | 3 (4.5%) |  | 73 (7.8%) | 22 (10.8%) |  |
| I do not know^3^ | 273 (24.0%) | 271 (25.4%) | 2 (3.0%) |  | 254 (27.2%) | 19 (9.4%) |  |
| **Are you in favor of alleviating legal restrictions on medical use of cannabis in France?** |  |  |  | 0.009^11^ |  |  | 0.045 |
| No | 81 (7.1%) | 80 (7.5%) | 1 (1.5%) |  | 71 (7.6%) | 10 (4.9%) |  |
| Yes | 845 (74.4%) | 785 (73.4%) | 60 (89.6%) |  | 680 (72.9%) | 165 (81.3%) |  |
| Did not adopt a position | 210 (18.5%) | 204 (19.1%) | 6 (9.0%) |  | 182 (19.5%) | 28 (13.8%) |  |
| **Are you in favor of alleviating legal restrictions on non-medical use of cannabis in France?** |  |  |  | <0.001 |  |  | 0.112 |
| No | 373 (32.8%) | 364 (34.1%) | 9 (13.4%) |  | 319 (34.2%) | 54 (26.6%) |  |
| Yes | 477 (42.0%) | 430 (40.2%) | 47 (70.1%) |  | 383 (41.1%) | 94 (46.3%) |  |
| Did not adopt a position | 286 (25.2%) | 275 (25.7%) | 11 (16.4%) |  | 231 (24.8%) | 55 (27.1%) |  |

IQR, interquartile range; GAD, general anxiety disorder; PHQ, patient health questionnaire.

^1^ Includes cannabis-cannabidiol co-users.

^2^ Chi² test was used for categorical variables and Mann-Whitney test for continuous variables (unless otherwise specified).

^3^ Modalities excluded for Chi-square or Fisher’s exact tests because of too low a number in cells.

**^4^** Generalized Anxiety Disorder scale-2,GAD-2 (34)

**^5^** Patient Health Questionnaire, PHQ-2 (35).

^6^ Item adapted from the Non-Motor Symptoms Scale for Parkinson’s Disease (36–38).

^7^ Item adapted from the Parkinson’s Disease Composite Scale (39,40).

^8^ Items adapted from the Graded Chronic Pain Scale-Revised (41)

**^9^** Rated according to the two previous questions (41)

**^10^** Item taken from the Pittsburgh Sleep Quality Index (42)

^11^ Fisher’s exact test

**^12^** Scoring based on the correctness of four ad hoc questions
