# Supplementary material for: Patterns of Use and Patient-Reported Effects of Cannabinoids in People With PD: A Nationwide Survey
Source: Parkinsons Dis. 2025 May 28;2025:2979089. doi: 10.1155/padi/2979089 (PMC12136873; doi:10.1155/padi/2979089)
Supplement: Supporting Information 4 — Supporting Table 3. Factors associated with cannabis and cannabidiol use (multivariable binary regression models). [file 2979089.f4.docx]

**Supplementary Table 3. Factors associated with cannabis and cannabidiol use (multivariable binary regression models)**

|  | **Cannabis** | | **Cannabidiol** | | **Cannabis and/or cannabidiol** | |
| --- | --- | --- | --- | --- | --- | --- |
|  | **aOR [95% CI]** | **p-value** | **aOR [95% CI]** | **p-value** | **aOR [95% CI]** | **p-value** |
| **Professional situation** |  |  |  |  |  |  |
| Retired | 1 |  |  |  |  |  |
| Working | 2.53 [1.36-4.74] | 0.004 |  |  |  |  |
| Other (including occupational disability) | 2.53 [1.33-4.80] | 0.005 |  |  |  |  |
| **“Presently, would you say that in your household, financially speaking…?”** |  |  |  |  |  |  |
| You are ok / You are comfortable | 1 |  | 1 |  | 1 |  |
| You just get by | 1.29 [0.70-2.37] | 0.410 | 1.22 [0.82-1.82] | 0.316 | 1.18 [0.80-1.74] | 0.406 |
| It’s difficult to make ends meet / You can’t manage without going into debt | 2.37 [1.17-4.79] | 0.016 | 1.74 [1.02-2.97] | 0.044 | 2.04 [1.22-3.41] | 0.006 |
| **Receiving deep brain stimulation** |  |  |  |  |  |  |
| No |  |  | 1 |  | 1 |  |
| Yes |  |  | 0.39 [0.18-0.81] | 0.012 | 0.43 [0.22-0.85] | 0.015 |
| **“Over the past three months, what number best describes your level of pain on average (0-10)?” *Per one unit increase*** |  |  | 1.08 [1.01-1.15] | 0.016 | 1.06 [1.00-1.13] | 0.041 |
| **Cannabinoid knowledge (0-4) ^1^ *Per one unit increase*** | 1.53 [1.23-1.91] | <0.001 | 2.29 [1.96-2.68] | <0.001 | 2.29 [1.97-2.66] | <0.001 |

**^1^** Scoring based on the correctness of 4 ad hoc questions
