# Supplementary material for: Patterns of Use and Patient-Reported Effects of Cannabinoids in People With PD: A Nationwide Survey
Source: Parkinsons Dis. 2025 May 28;2025:2979089. doi: 10.1155/padi/2979089 (PMC12136873; doi:10.1155/padi/2979089)
Supplement: Supporting Information 5 — Supporting Table 4. Frequency and duration of use for cannabis and cannabidiol. [file 2979089.f5.docx]

**Supplementary Table 4. Frequency and duration of use for cannabis and cannabidiol**

|  | **Cannabis (n=67)**  **%** | **Cannabidiol (n=203)**  **%** |
| --- | --- | --- |
| **Frequency of use** |  |  |
| < once a week | 52.2 | 38.9 |
| Once a week | 6.0 | 10.3 |
| > Once a week and < once a day | 17.9 | 17.7 |
| Once a day | 11.9 | 18.2 |
| Twice a day | 4.5 | 8.4 |
| ≥ three times a day | 7.5 | 6.4 |
| **Regular use for^1^** |  |  |
| < a year | 34.4 | 66.4 |
| ≥ a year | 65.6 | 33.6 |

**^1^** Regular use was characterized as a frequency of use ≥ once a week (n=32 and n=122 for cannabis and cannabidiol, respectively)
